# Supplementary material for: Silk/Natural Rubber (NR) and 3,4-Dihydroxyphenylalanine (DOPA)-Modified Silk/NR Composites: Synthesis, Secondary Structure, and Mechanical Properties
Source: Molecules. 2020 Jan 6;25(1):235. doi: 10.3390/molecules25010235 (PMC6982767; doi:10.3390/molecules25010235)
Supplement: Supplementary file 1 [file molecules-25-00235-s001.pdf]

Silk/natural rubber (NR) and 3,4-dihydroxyphenylalanine (DOPA)-modified silk/NR composites: synthesis, mechanical properties and secondary structures

Hiromitsu Sogawa[1], Treratanakulwongs Korawit[1], Hiroyasu Masunaga[2] and Keiji Numata[1],*

1 Biomacromolecules Research Team, RIKEN Center for Sustainable Resource Science, 2-1, Hirosawa, Wako-shi, Saitama, 351-0198

2 Materials Structure Group I, Research & Utilization Division, Japan Synchrotron Radiation Research Institute, 1-1-1, Kouto, Sayo-cho, Sayo-gun, Hyogo 679-5198, Japan

* Correspondence: keiji.numata@riken.jp; Tel.: +81-48-467-9525; Fax: +81-48-462-4664

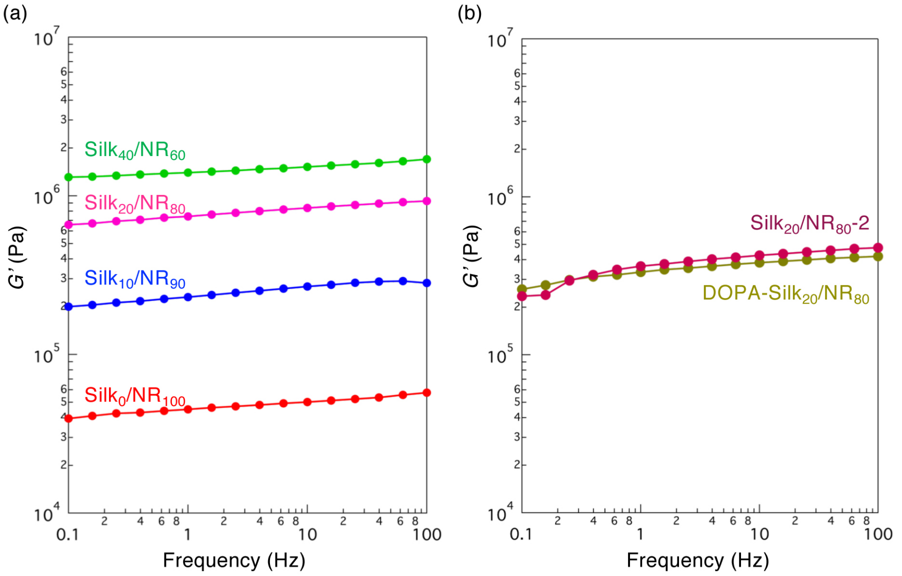

Figure S1. The frequency dependency of (a) silk/NR with different silk contents and (b) DOPA-silk$_{20}$/NR$_{80}$ and its control (silk$_{20}$/NR$_{80}$-2). The strain was fixed at 0.1%.

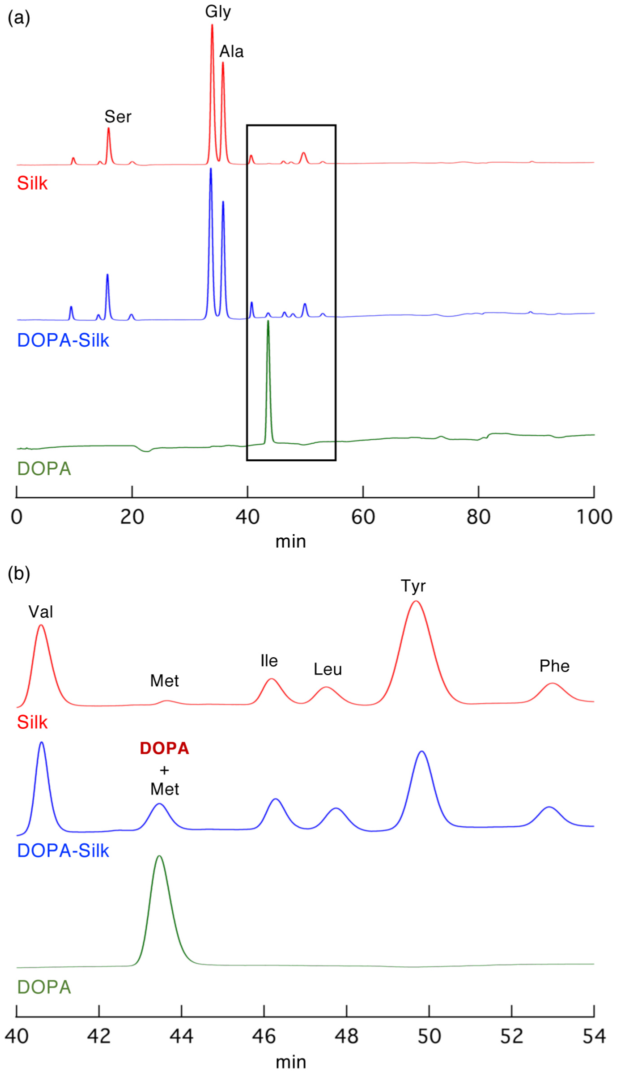

Figure S2. Amino acid analysis data by HPLC. Peaks for pure (unmodified) silk solution, DOPA-modified silk solution, and DOPA (control) in the range of (a) 0–100 min and (b) 40–54 min. Although the methionine peak, which was originally included in silk from *B. mori* cocoons, overlapped with the DOPA peak, the peak ratio of methionine was subtracted appropriately from DOPA-silk to properly determine the DOPA content.

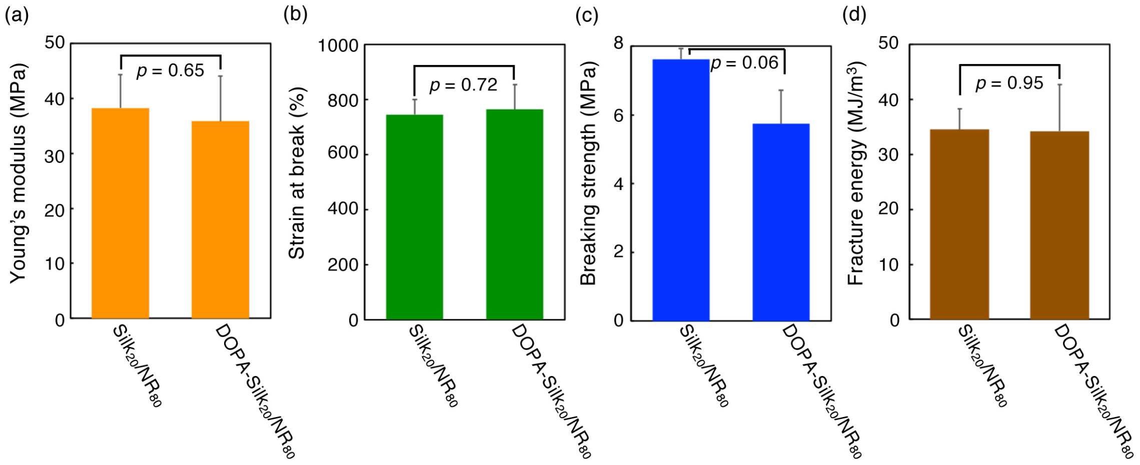

Figure S3. Mechanical properties of silk$_{20}$/NR$_{80}$ and DOPA-silk$_{20}$/NR$_{80}$: (a) Young's modulus, (b) strain at break, (c) breaking strength, and (d) fracture energy. No significant differences were found between these groups. The p-values between groups were described for each graph.

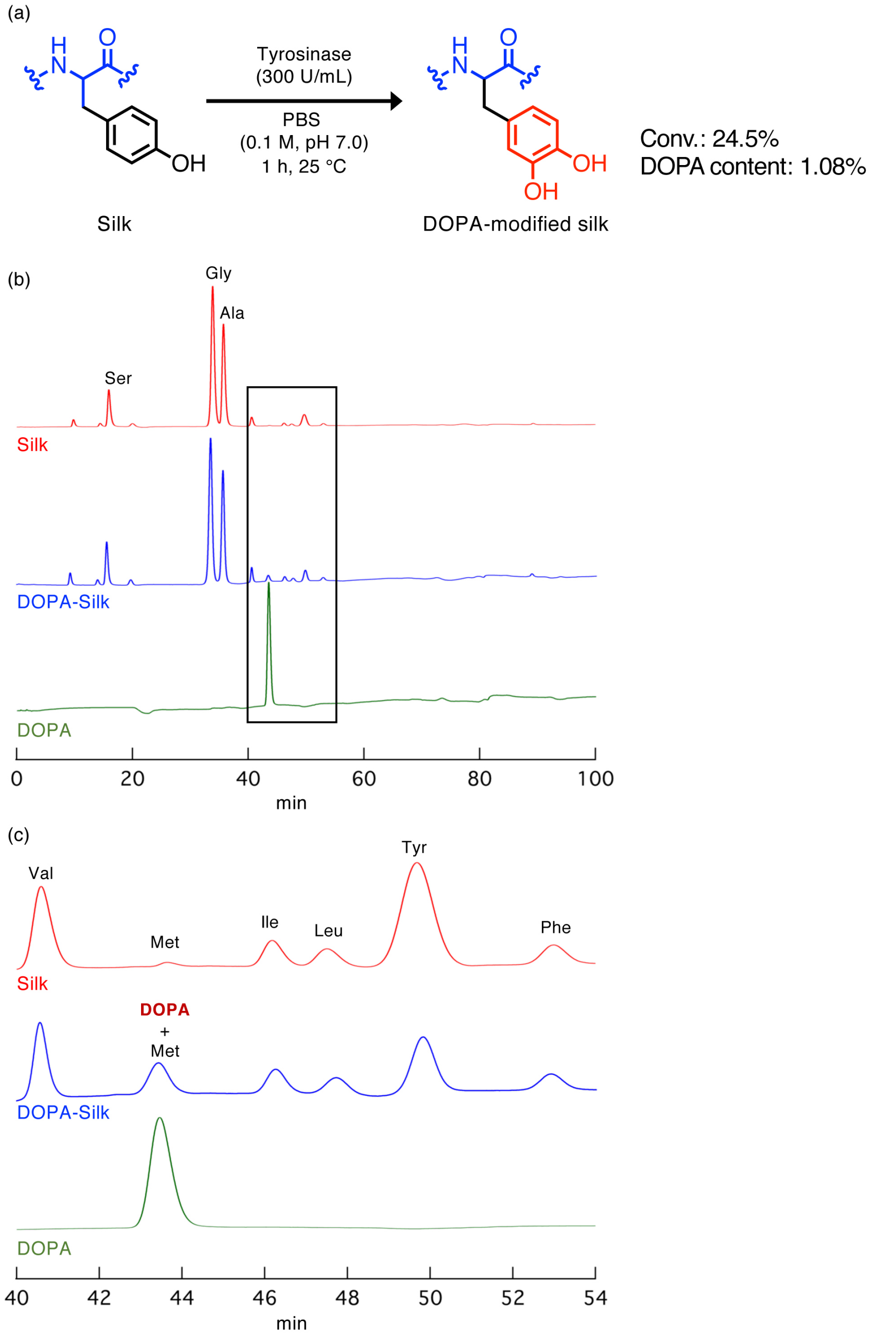

Figure S4. (a) Tyrosinase-catalyzed DOPA modification of silk. Silk solution was mixed with tyrosinase (300 U/mL) and PBS (0.1 M, pH 7.0) and stirred for 1 h at 25 °C. The reaction conversion and DOPA content were determined by below amino acid analysis data by HPLC. Peaks for pure (unmodified) silk solution, DOPA-modified silk solution, and DOPA (control) in the range of (b) 0–100 min and (c) 40–54 min. Although the methionine peak, which was originally included in silk from *B. mori* cocoons, overlapped with the DOPA peak, the peak ratio of methionine was subtracted appropriately from DOPA-silk to properly determine the DOPA content.
